# On the Use of the Potassa Fusa in the Local Treatment of Boils and Carbuncles

**Published:** 1856-02

**Authors:** Benjamin Travers


*On the use of the Potassa Fusa in the Local Treatment of Boils and Carbuncles.* By BENJAMIN TRAVERS, Esq., Jun.

A succession of well-marked instances of the great advantage to be obtained by the use of a powerful caustic like the "kali purum," in securing the safe and far more speedy convalescence of those who are suffering under the above disorders, at any period of life, induces me to request that you will give insertion to the following remarks in an early number of your journal. It is only of late, and, indeed, even now but to a very limited extent, that the Surgeons of this country have begun to appreciate the great superiority of this method. This is not surprising, when we see to what an extent the idea has been discouraged by writers even of our own day. Their objections, however, are unreasonable and unscientific, and they have been advanced by those who knew nothing whatever of the effects of this practice in an experimental point of a view, or as a matter of personal experience. In what has been termed the second stage of these collections, provision must be made by means of an artificial opening for the escape of the dead matters beneath the strained and spoiled integument, which in its turn must surely die, albeit more slowly, and to a more limited extent. It should be borne in mind, that we are not here dealing with a texture which has undergone the attenuation consequent upon an interstitial removal, but that the parts are already condemned, and will infallibly die to a limited extent, since they are the seat of a mode of inflammation having a very different event from that which determines the progress of a common acute abscess. Such a collection, when ripe, we prick or cut, in strict submission to and observance of a natural law. For an equally good reason we ought not to cut boils and carbuncles. For the present I must not enlarge on this head, but content myself with observing, that besides the anomaly implied in cutting and wounding a mass of inflamed tissue already disposed to die from excessive irritation, the risk of a fatal hæmorrhage so incurred is by no means an imaginary accident. The subsequent low rate of healing after the use of the knife constitutes another most grave objection to its employment. Dr. Physick, of Philadelphia, has, in my opinion, established an undivided claim to the merit of having first used the caustic potash, with a scientific view, in the treatment of carbuncle. (Vid. Philadelphia Journal of Medical and Physical Science, Vol. II.) He was the friend and pupil of John Hunter, and certainly the most distinguished man of his time in America. He died so lately as the year 1837, and has been called the Father of American Surgery. (Vid. "A Memoir," etc., by J. Randolph, M. D., Philadelphia, 1839.) Dr. Physick says, "that the suffering ceases as soon as the pain of the caustic has subsided." I cannot do justice to the importance of this observation in a communication like the present, but I possess, and, indeed, am constantly accumulating fresh evidence of its truth, both as regards boil and carbuncle. M. Maunoir, of Geneva, the celebrated Ophthalmic Surgeon, strongly advocated this practice. Indeed it needs but a patient trial to convince the least credulous of its extraordinary efficacy. I shall now append a brief notice of four cases demonstrative of the fact, that a corrosive caustic possesses many decided advantages over the knife, in the local treatment of these maladies.

Mr. Travers relates four cases in which the caustic was most successfully applied by him, after which he remarks:—

I find from a report, which I lately read in a newspaper, that, in 1854, there occurred ninety deaths from "Carbuncle" in the metropolis alone. It is notorious that this and other allied local actions are more frequent and more serious than they used to be ten years ago.—*Medical Times and Gazette.*

